# Confirmation of highly pathogenic avian influenza H5N1 in skuas, Antarctica 2024

**DOI:** 10.3389/fvets.2024.1423404

**Published:** 2024-12-06

**Authors:** Benjamín Bennett-Laso, Bárbara Berazay, Gabriela Muñoz, Naomi Ariyama, Nikita Enciso, Christina Braun, Lucas Krüger, Miloš Barták, Marcelo González-Aravena, Victor Neira

**Affiliations:** ^1^Departamento de Medicina Preventiva Animal, Facultad de Ciencias Veterinarias y Pecuarias, Universidad de Chile, Santiago, Chile; ^2^Programa de Doctorado en Ciencias Silvoagropecuarias y Veterinarias, Universidad de Chile, Santiago, Chile; ^3^Programa de Magister en Ciencias Animales y Veterinarias, Universidad de Chile, Santiago, Chile; ^4^Polar & Bird Ecology Group, Institute of Ecology and Evolution, Friedrich Schiller University Jena, Jena, Germany; ^5^Instituto Antártico Chileno, Punta Arenas, Chile; ^6^Millennium Institute of Biodiversity of Antarctic and Subantarctic Ecosystems (BASE), Santiago, Chile; ^7^Department of Experimental Biology, Masaryk University, Faculty of Science, Brno, Czechia

**Keywords:** highly pathogenic avian influenza, avian influenza, Antarctica, Antarctic wildlife, skuas, H5N1 2.3.4.4b

## Abstract

From December 2023 to March 2024, a surveillance program aiming to detect Highly Pathogenic Avian Influenza (HPAI) H5N1 was conducted on Antarctica territories, specifically at Fildes Peninsula (King George Island, Maritime Antarctic), and James Ross Island. At Fildes Peninsula, samples from marine birds and mammals were collected from four accessible sampling locations with significant animal colonies: Ardley Island, hosting a large concentration of Gentoo penguins (*Pygoscelis papua*); Ardley Cove, where small groups of likely non-breeding Chinstrap penguins (*Pygoscelis antarcticus*) were present; seal haul-out sites of Southern elephant (*Mirounga leonina*) and Weddell (*Leptonycotes wedellii*); and, a nesting site of Southern giant petrels (*Macronectes giganteus*). Additionally, six samples were collected from five dead skuas near the Lachman lakes on James Ross Island (63.7989S, 57.8105W) on March 3, 2024. Despite collecting a total of 943 samples from Fildes Peninsula, all results tested negative for HPAI, and no animals displayed clinical signs or behaviors consistent with HPAI infection. However, all skua samples from James Ross Island tested positive for HPAI H5N1 clade 2.3.4.4 by specific real-time RT-PCR reactions, confirming the first recorded HPAI-related mortality event in Antarctica (south of 60°S), specifically in skuas. Further research is necessary to genetically characterize the virus and better understand the role of skuas in viral dissemination in Antarctica.

## 1 Introduction

Highly pathogenic avian influenza (HPAI) subtype H5NX, clade 2.3.4.4b has been responsible for numerous global outbreaks, leading to multiple mass mortality events in avian wildlife and marine mammal populations. Human cases of this virus have also been reported, underscoring its zoonotic potential and public health risk (1–3). Additionally, HPAI infections may significantly stress breeding seabirds, potentially resulting in breeding failure or death due to increased energy demands (4, 5). The rapid and widespread dissemination of this virus is primarily driven by infected migratory species (6–8), particularly those with long-range dispersal capabilities (9, 10). The current HPAI H5N1 strain, clade 2.3.4.4b, emerged in 2020 after extensive viral evolution and was first confirmed in South America in October 2022 (11). Prior to this, the virus had not been detected in Oceania (Australia and New Zealand) or Antarctica.

Given the close proximity of South America to Antarctica, coupled with the migratory patterns of seabirds between the two regions, concerns have risen about the virus’s potential introduction to Antarctica via migratory birds. Previous evidence suggests that influenza viruses detected in Antarctica are genetically related to strains found outside the continent (12, 13). Migratory species such as Arctic terns (*Sterna paradisaea*) (14) and South polar skuas (*Stercorarius maccormicki*) (15) are known to undertake trans-equatorial migrations, with both species demonstrating the ability to shed the virus. With the arrival of the virus in the southern hemisphere, other species with shorter migration patterns and wide foraging areas, such as giant petrels (*Macronectes* spp.), and Brown skuas (*Stercorarius antarcticus lonnbergi*) may play a more significant role in viral spread (16), potentially introducing the virus to Antarctic wildlife before developing visible clinical signs. Although some positive cases of HPAI H5NX 2.3.4.4b have been confirmed on South Georgia island, a Sub-Antarctic territory (17), no published cases have been reported in more southern latitudes. This suggests that Antarctic species may be highly susceptible to infection, making them vulnerable to mass mortality events if the virus spreads to Antarctic colonies (18). To investigate this risk, we conducted surveillance for HPAI in seabirds and marine mammals at key site on King George Island (South Shetland Islands) and the Maritime Antarctic Peninsula, areas close to previously confirmed cases and where wildlife-human interaction is increasing (19, 20). We also coordinated with researchers across the Antarctic Peninsula to receive reports and samples from unexpected mortality events. In response, a Czech Antarctic team alerted us to dead seabirds near Lachman Lakes on James Ross Island (63.7989 S, 57.8105 W), 4 km east of Mendel Base. This report summarizes findings from December 2023 and March 2024, confirming the presence of HPAI H5N1.

## 2 Materials and methods

### 2.1 Locations at Fildes Peninsula, King George Island

The surveillance team was based at Professor Julio Escudero Base, located on King George Island, in the South Shetland Islands, Maritime Antarctic (33). From December 16, 2023, to March 23, 2024, regular surveillance expeditions were carried out around the base (see Supplementary Figure S1), covering key wildlife areas with high concentrations of penguins, flying seabirds, and marine mammals. During these expeditions, clinical observation and sample collections were performed. The selected locations were based on known animal aggregations and colony sites, as detailed by environmental monitoring studies (21). Ten specific sites were visited at least once (see Supplementary Figure S1), including Ardley Cove, Ardley Island (ASPA 150), Hydrographers Cove, Diomedea Island, Flat-top Peninsula, Biologists Cove, Basalt Creek, Gradzinski Cove, and Green Point. These locations were selected based on accessibility and the presence of notable species, such as Gentoo penguins (*Pygoscelis papua*), Chinstrap penguins (*Pygoscelis antarcticus*), Weddell seals (*Leptonychotes weddellii*), Southern giant petrels (*Macronectes giganteus*), Southern elephant seals (*Mirounga leonina*) and others (Supplementary Table S1). The sampling effort was recorded by tracking researchers’ movements using a Garmin InReach Explorer and a Garmin Enduro Watch device, which recorded geographic positions at 60- and 30-s intervals, respectively. The tracking data was processed using the “trip” R-package (22) to quantify time spent at different sites, while ArcMap was used to visualize the distribution of effort. Additionally, veterinarians conducted clinical observations, monitoring for signs of respiratory, neurologic, or digestive syndromes associated with HPAI H5N1. Sampling was performed on live and dead animals in compliance with the guidelines of the University of Chile’s Institutional Ethics Committee (approval no. 22603-VET-UCH).

### 2.2 Sampling

Sampling activities were conducted under permission numbers 669–2023, 670–2023, and 201–2024 from the Chilean Antarctic Institute INACH. Field researchers used personal protective equipment (PPE), including coveralls, gloves, face shields, and masks, which were disinfected and safely disposed of at the sampling site. Afterward, the sealed waste was transported to Professor Julio Escudero Base for proper disposal, minimizing the risk of viral spread. Researchers focused on collecting fresh fecal samples to reduce animal handling and increase the number of samples obtained. In some cases, direct samples were collected from live Southern giant petrels, skuas, and opportunistically from freshly deceased animals. Sampling tools included rayon swabs, which were placed into Viral Transport Media (Inactivated Type) (ALLTEST catalog number ITM-001). For environmental samples, pooled groups of five swabs were placed in a single tube of viral transport media. Direct samples from cloacal swabs were collected by capturing and restraining individuals according to pre-approved protocols (069/CEC/2018 and 3/CBSCUA/2022). All procedures adhered to Antarctic Treaty guidelines, and the field team consisted of a biologist and four veterinarians who also monitored the animals for clinical signs of disease. No blood samples were collected, as there was no prior evidence of HPAIV in King George Island populations.

### 2.3 Unexpected mortality case at James Ross Island

**Figure 1:**
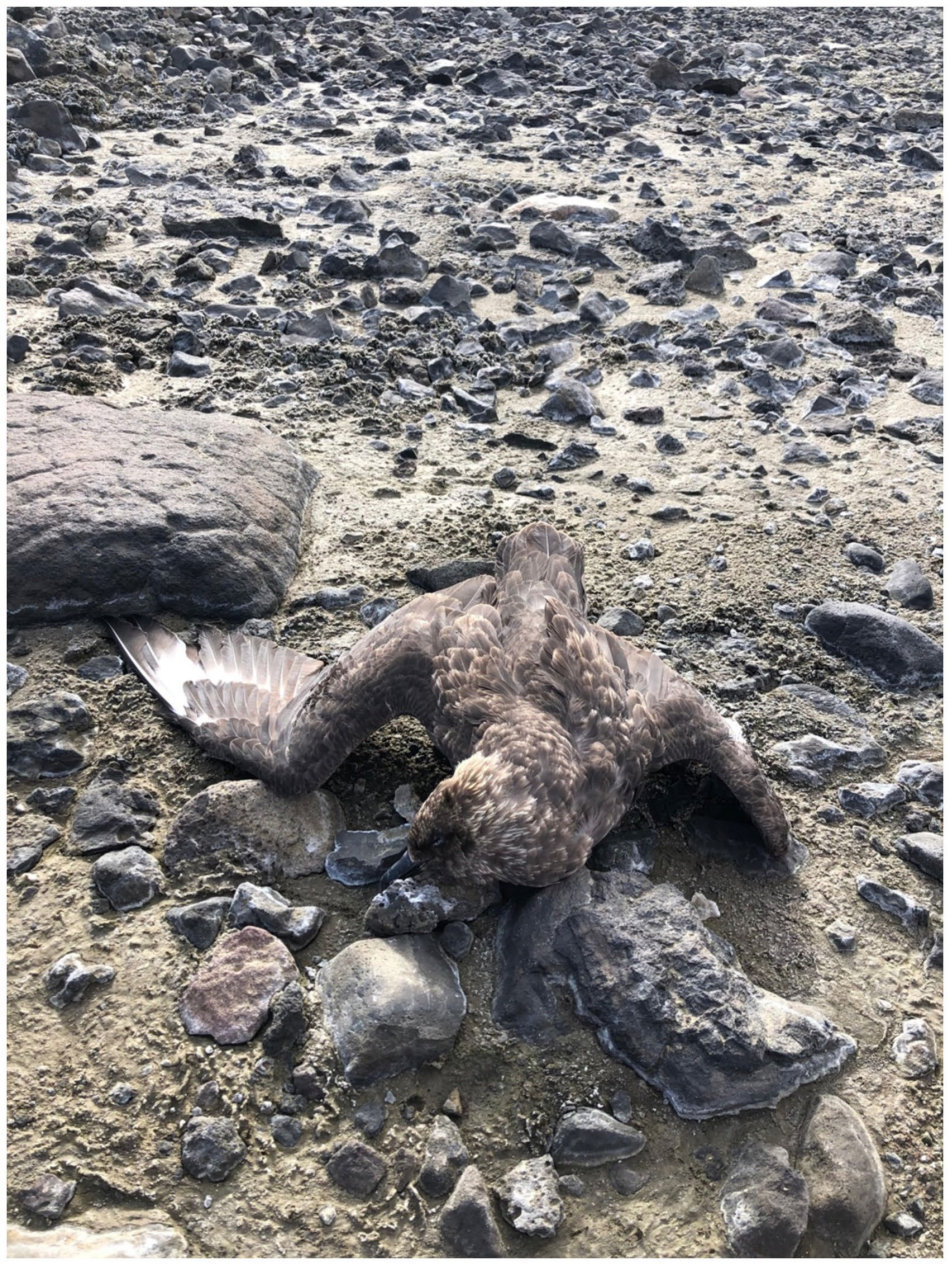
Deceased skua (brown skua) at James Ross Island.

On February 28, 2024, a report of three dead skuas (unidentified species) was received from a field crew working with the Czech Antarctic Research Program near the Base Johann Gregor Mendel (63.7989S, 57.8105W) on James Ross Island. The site, a known nesting area for skuas, is adjacent to the freshwater Lachman lakes. The researchers, who had over 20 years of experience working in the region, indicated that finding multiple dead skuas in one location was unprecedented. Our surveillance team traveled from King George Island to James Ross Island in the Chilean vessel *Janequeo* (ATF-65), arriving on March 3, 2024. At the site, five dead skuas were found, and samples were collected from all individuals. Swabs from tissues were pooled from different organs, following established protocols. One skua was positively identified as a brown skua (Figure 1), though the state of decomposition and developmental stages made species identification of the other individuals more challenging.

### 2.4 Diagnostic testing

The collected samples were processed within 24 h after collection at the molecular biology laboratory at Professor Julio Escudero Base. RNA extraction was performed using the acid guanidinium thiocyanate-phenol-chloroform method with Chomczynski phenol solution (Winkler Ltda.), following the manufacturer’s instructions. A real-time reverse transcriptase polymerase chain reaction (RT PCR) assay performed targeting the conserved M gene of the influenza virus was conducted using the primers infA F (5’-GACCRATCCTGTCACCTCTGAC-3′), infA R (5’-AGGGCATTYTGGACAAAKCGTCTA-3′), infA Probe1 (5’ FAM-TGC AGT CCT CGC TCA CTG GGC ACG-BHQ1-3′) (11). Samples with cycle threshold (CT) values greater than 35 were considered inconclusive, while those with CT values greater than 40 were deemed negative for influenza virus. Positive samples were further subtyped and pathotyped to confirm HPAI H5N1 clade 2.3.4.4 using specific protocols from the NVSL (protocols 1732.02, 1767.01, and 1768.01) (11, 23).

## 3 Results

Our observation and sampling efforts on Fildes Peninsula totaled 141 h and 30 min, covering 209.5 km of transects (Supplementary Figure S1). The highest efforts were at Diomedea Island (25.4 h), Ardley Island (22.7 h), Biologists Cove (10.8 h), Gemel Peaks (9.2 h), and Gradzinski Cove (7.4 h). We observed various species, including Gentoo, Chinstrap, and Adélie penguins, Southern giant petrels, Antarctic shags, Antarctic terns, Brown and South polar skuas, Kelp gulls, Antarctic fur seals, and non-breeding Southern elephant, Weddell, and Leopard seals. None showed clinical signs of HPAI, and no unexpected mortality was detected. However, skua populations, particularly South polar skuas, declined compared to previous years. One skua carcass was found near Ardley Island, along with a few Gentoo penguin chick carcasses, consistent with typical mortality. In late February, 15 Chinstrap penguin carcasses were observed at Elephant’s Beach, all testing negative for HPAI. In total, 943 samples were collected, 857 environmental fecal samples and 88 direct samples, all testing negative for Influenza A by real-time RT-PCR.

The real-time RT-PCR testing results for skuas from James Ross Island are detailed in Table 1. All deceased skuas tested positive for HPAI H5N1 clade 2.3.4.4, with CT values ranging from 13 to 20.7, indicating high viral loads. There was no evidence of predation or scavenging on the dead animals. Although we lacked permission to histopathological analysis, evident macroscopic lesions were not observed. Additionally, two healthy skuas were noted in proximity to the deceased individuals.

**Table 1:** Real-time RT-PCR cycle threshold (ct) values from suspected skuas at James Ross Island.

ID	Sample	Species	Gene M ct	H5 2.3.4.4 ct	HPAI*ct	N1 ct
1	Brain-Lung	Skua spp	16.4	15.4	13	16.6
2	Brain-Lung	Skua spp	18.6	18	16.1	18.8
3	Brain-Lung	Skua spp	19.4	18.3	17.5	20.7
4	Brain	Skua spp	18.8	17.6	15.8	18.7
5	Brain-Trachea	Skua spp	16.7	15.5	13.9	16.5

All samples were confirmed positive for HPAI H5N1 2.3.4.4. *HPAI pathotyping.

## 4 Discussion

During the 2022–2023 Southern Hemisphere spring and summer, HPAI outbreaks were confirmed across several South American countries, bringing the virus closer to Antarctica (4). Before this report, HPAI had been detected in sub-Antarctic regions, including South Georgia and the Falkland Islands (17). Then, this study aimed to monitor HPAI in marine mammals and seabirds at key sites like Fildes Peninsula on King George Island, where human activity increases the risk of viral spread (24). Additionally, our network with Antarctic researchers facilitated rapid reporting of unexpected mortality events.

Our surveillance confirmed the presence of HPAI H5N1 in a mortality event involving skuas at James Ross Island. Specific TaqMan PCR assays confirmed the H5 subtype, clade 2.3.4.4, as well as the N1 subtype. Although further genetic analysis and sequencing are necessary, these results provide timely and crucial evidence of the virus’s presence in Antarctica. The reliability of multiple TaqMan PCR reactions enhances confidence in these findings.

The mortality event on James Ross Island was significant, as dead skuas are rarely observed in large numbers. The low CT values from the samples suggest that HPAI H5N1 was the likely cause of death. This finding aligns with recent reports of the virus in skuas within Antarctic territories. On February 25, 2024, just days before the James Ross Island case, two skuas at the Primavera Antarctic Base on Cape Primavera (64°09′00 ″S 60°57′50″W) were confirmed positive for HPAI H5N1, though the report has yet to be published. Additionally, (34) reported suspected cases of HPAI affecting Adélie penguins and Antarctic shags at Red Rock Ridge, though those animals exhibited no clinical signs. These suspected cases remain tentative due to reliance on conventional PCR assays and also raised questions about their accuracy (25).

In contrast, no signs of HPAI were observed in the wildlife of Fildes Peninsula. The extensive sampling and negative RT-PCR results suggest that HPAI was not present at this site during our surveillance period. The decline in the skua population, particularly among South polar skuas, raises questions about the virus’s impact. It is possible that infected birds died during migration, never reaching their breeding grounds in Antarctica. The absence of mass mortality events among other species in the region could indicate a slower viral spread or lower transmission rates in Antarctic ecosystems.

The confirmation of HPAI H5N1 in skuas highlights their potential susceptibility to the virus, which could explain the population decline at Fildes Peninsula. Previous studies have documented the susceptibility of Great Skuas to HPAI H5N1, with a massive mortality event occurring on Foula Island, United Kingdom (26, 27). However, as top predators and scavengers, skuas may have a lower risk of rapid virus dissemination compared to densely populated species like penguins. The study of skua species, including serological analysis, is essential for understanding their role in HPAI dynamics in Antarctica.

Other migratory species, such as Southern giant petrels and Kelp gulls, may also contribute to HPAI dissemination due to their movements between temperate, sub-Antarctic, and Antarctic waters. These species, known for scavenging behaviors, have been implicated in the spread of low-pathogenicity avian influenza viruses (LPAIV) in previous studies (12, 13, 28, 29). While Kelp gulls and Snowy sheathbills have been suggested as potential vectors, tracking studies to confirm their migratory movements are lacking. The role of migratory marine mammals, particularly male Southern elephant seals, in HPAI transmission, also cannot be excluded.

The arrival of HPAI H5N1 in Antarctica presents a significant risk to its wildlife. Outbreaks in South Africa, Chile, and Argentina have demonstrated the high susceptibility of Spheniscus penguins (30, 31). Although no mass mortality events involving penguins have been reported in Antarctica, a recent massive mortality event in Argentina resulted in the deaths of hundreds of Southern elephant seal pups due to HPAI H5N1 (32).

Although sequencing data is pending, our findings are highly reliable, supported by consistent clinical signs and multiple TaqMan PCR detections. This data holds particular value, especially as no other peer-reviewed reports have confirmed the virus in Antarctica to date. The detection of HPAI H5N1 in skuas at James Ross Island, despite the absence of clinical signs in Fildes Peninsula wildlife, indicates that viral transmission in Antarctic species may be slower and less widespread than in other regions. Future studies focus on key species like skuas to fully understand the virus’s evolution and transmission in this fragile ecosystem.

## Data Availability

The datasets presented in this study can be found in online repositories. The names of the repository/repositories and accession number(s) can be found in the article/Supplementary material.
