# Supplementary material for: Confirmation of highly pathogenic avian influenza H5N1 in skuas, Antarctica 2024
Source: Front Vet Sci. 2024 Dec 6;11:1423404. doi: 10.3389/fvets.2024.1423404 (PMC11660801; doi:10.3389/fvets.2024.1423404)
Supplement: Supplementary file 1 [file Data_Sheet_1.pdf]

Supplementary Figure 1. Location of the Escudero Research Station (red star) and James Ross Island (blue star) on the scale of the Antarctic Continent (a), on the scale of the South Shetland Islands (b) and Fildes Peninsula in detail (c) with the cumulative effort calculated from tracking researcher movements during sampling and surveillance for symptomatic animals with clinical signs compatible with high pathogenic avian influenza. Names of locations in ‘c’ from the Composite Gazetteer of Antarctica, Secretariat SCAR https://data.aad.gov.au/aadc/gaz/scar/download.cfm. Seal samples were taken in Hydrographers Cove, Flat-top Peninsula, Biologists Cove, Basalt Creek, and Gradzinski Cove; penguin samples were taken from Ardley Cove, Ardley Island, and Hydrographers Cove; giant petrel samples were taken from Diomedea Island (Petrels’ Islet); breeding shag samples were taken in Ardley Island; Black ´x´ are location of Skuas’ samples in Ardley Island, Hydrographers Cove, and Gemel Peaks. See Supplementary Table 1 for details on samples taken. Further fauna observations (without sampling) were done through every covered area, emphasizing 78 Southern Giant Petrel active nests in the upper terrains of Gradzinski Cove and 52 active nests in Diomedea Island; two skua nests between Halfthree Point and Geographers Cove and two nests in the upper terrains of Gradzinski Cove; Elephant, Weddell, and Fur Seals in Geographers Cove, Flat-top Peninsula, Green Point, and a small group of breeding Fur Seals in Gradzinski Cove; one Leopard Seal and Breeding Kelp Gulls in Green Point; Chinstrap Penguins in Ardley Cove, Hydrographers Cove, and Basalt Creek.

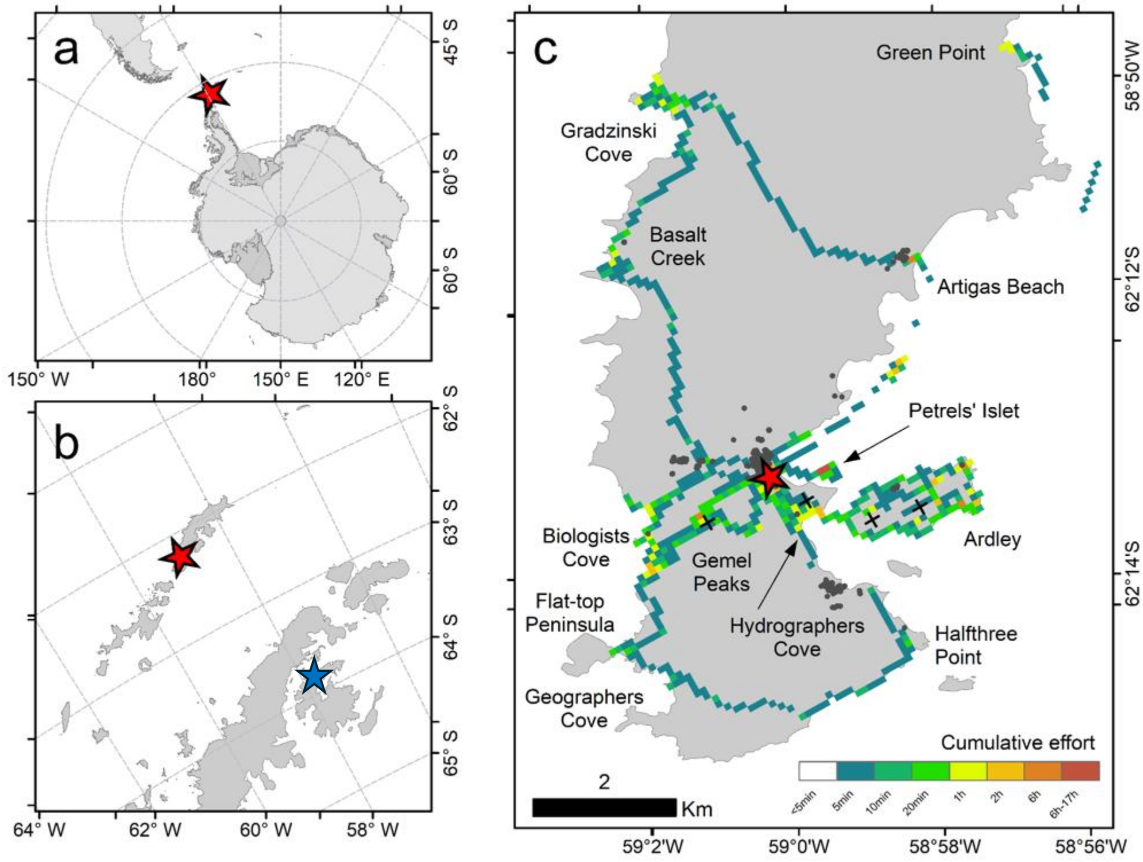

Supplementary Table 1. Sampling details during high pathogenic avian influenza (HPAI) surveillance Sampled locations, species, types of samples (environmental feces swabs and direct cloacal swabs), and quantities. Positive samples were collected only from James Ross Island.

Locations	Species	Environmental	Direct	Total
**Ardley Island**	Gentoo penguin	725 (145 pools of 5 fecal samples)	2 (brain sample)	727
	Adelie penguin	40 (8 pools 5 fecal samples)	-	40
	Antarctic Shag	-	4 (cloacal swab)	4
	Chinstrap Penguin	-	3 (cloacal swab)	3
	Brown Skua	2	1	3
**Hydrographers Bay, King George Island**	Gentoo penguin	-	2	2
	Weddell seal	8	-	8
	Chinstrap penguin	-	9 (cloacal swab)	9
	Brown Skua	-	1 (brain sample)	1
	Emperor penguin	-	1 (cloacal swab)	1
**Gemel peaks**	Brown Skua	-	2 (cloacal swab)	2
**Diomedea Island (Petrel's Islet)**	Southern Giant Petrel	-	39 (cloacal swab)	39
	Unidentified	1	-	1
**Elephant's beach**	Chinstrap Penguin	-	23 (brain and lung samples)	23
	Elephant seal	29	-	29
	Weddell seal	4	-	4
	Fur seal	2	-	2
	Gentoo penguin	5 (1 pool of 5 fecal samples)	-	5
	Unidentified	1	-	1
**Basalt Creek and Gradzinski Cove**	Elephant seal	11	-	11
	Weddell seal	4	-	4
	Fur seal	3	-	3
**Ardley Cove**	Chinstrap penguin	5 (1 pool of 5 fecal simples)	-	5
	Gentoo penguin	5 (1 pool of 5 fecal samples)	1 (cloacal swab)	6
**Collins Base**	Antarctic gull	10	-	10
**James Ross Island**	Skua spp	-	6 (tissue pool samples)	6
**Total**		855	94	949
